# Entropy SVM–Based Recognition of Transient Surges in HVDC Transmissions

**DOI:** 10.3390/e20060421

**Published:** 2018-05-31

**Authors:** Guomin Luo, Changyuan Yao, Yinglin Liu, Yingjie Tan, Jinghan He

**Affiliations:** School of Electrical Engineering, Beijing Jiaotong University, Beijing 100044, China

**Keywords:** HVDC transmission, frequency spectrum entropy, SVM, transient surge recognition

## Abstract

Protection based on transient information is the primary protection of high voltage direct current (HVDC) transmission systems. As a major part of protection function, accurate identification of transient surges is quite crucial to ensure the performance and accuracy of protection algorithms. Recognition of transient surges in an HVDC system faces two challenges: signal distortion and small number of samples. Entropy, which is stable in representing frequency distribution features, and support vector machine (SVM), which is good at dealing with samples with limited numbers, are adopted and combined in this paper to solve the transient recognition problems. Three commonly detected transient surges—single-pole-to-ground fault (GF), lightning fault (LF), and lightning disturbance (LD)—are simulated in various scenarios and recognized with the proposed method. The proposed method is proved to be effective in both feature extraction and type classification and shows great potential in protection applications.

## 1. Introduction

High voltage direct current (HVDC) transmission plays an important role in power transmissions due to its advantages such as large transmission capacity and good performance in power flow control [1,2]. It has been widely applied in delivering large amount of power and connecting asynchrony power grids. Generally, traveling wave–based protection or voltage derivate based protection are used as the primary protection, and under-voltage protection or current-differential protection are adopted as the backup protections in the HVDC systems [3,4]. Traveling wave–based protection captures the transient traveling surges on transmission lines and make quick response in a very short time. As the shunt capacitors and the smoothing inductors in convertor station can effectively reflect traveling waves, traveling wave–based protection can easily distinguish faults beyond the protected zone. The time-domain features, such as magnitude and changing rate of electrical measurements, are commonly used in protection judgement [5,6].

However, the time domain–based method is sensitive to surge disturbances, for example, lightning strokes [7,8]. The transient waveforms of surge interferences look similar with the ones of ground faults in some cases. Such similarity makes them difficult to be discriminated. In order to improve the reliability of protection actions, the identification of transient surges is a critical function of protection algorithm, which includes two important aspects: feature extraction and classification algorithm.

To effectively identify transient surges, the unique features of various signals should be extracted, and the features should represent the signals with a stable and reliable performance. Frequency analysis that can provide more details on spectrum differences are often used to fully utilize the signal transient information in both time and frequency domains and reveal a better characterizing performance. Various frequency-based features are adopted and reported to generate good performance—for example, wavelet energy spectrum [9], S-transform distribution [10,11], and frequency energy spectrum [12]. These amplitude-based or energy-based features heavily depend on the magnitude of spectrum, which can be distorted during propagation. Entropy, which only describes the distribution of frequency spectrum, was proved to be effective in charactering transient surges in HVDC. Compared with traditional magnitude-based feature extractions that will be affected by unequally distortions, the entropy can characterize the distribution of energy in a certain range. It is a powerful tool to extract transient signal characteristics and is immune to variations of magnitude attenuations and distortions [13,14,15,16].

Besides good features, the classification algorithm is also an important factor in recognizing various transient signals. In most cases, a threshold is used to realize the signal classification, and the value of threshold is sometimes selected manually according to different signal characteristics. Such a threshold-based method is susceptible to the influence of line parameters, transition resistance, noise and so on [16,17]. Machine learning algorithms can effectively solve the problems of uncertain correspondence [18]. They show a number of advantages in pattern recognition, classification, and generalization and play an important role in the field of power system fault diagnosis [19,20,21]. A lot of classification algorithms are used in fault analysis, such as artificial neural networks (ANNs), support vector machines (SVMs), auto-encoders, expert systems, and so on [22,23,24]. The two most popular classifiers are ANN and SVM. When compared with SVMs, ANNs are reported to have slow algorithm convergence, poor adaptability, and high requirements for training samples. SVMs, which are proposed based on statistical learning theory, reveal better capability in solving classification problems with a small size of samples. For fault classification of power systems, where the number of samples are limited, SVM could be a better choice [25,26].

Combining the advantages of entropy and SVMs, this paper proposed a transient surge recognition method to improve the reliability of HVDC protections. Based on the analysis of transient waveforms of pole-to-ground faults (GFs), lightning faults (LFs), and lightning disturbances (LDs) in direct current (DC) transmission lines, the features of different transient signals are represented by frequency spectrum entropy (FSE) vectors. The FSE vector is then used as the input of a SVM structure to realize the recognition of transient interferences. A typical HVDC transmission line is modeled and simulated in different scenarios to demonstrate the effective performance of proposed method. The simulation results and the comparisons prove the potential of FES-SVM in protection applications.

## 2. HVDC and Transient Surges

### 2.1. Fundamentals of HVDC

**Figure 1:**
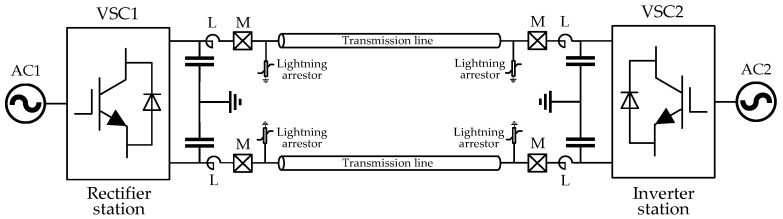
Diagram of a typical voltage-source converters (VSC)-high voltage direct current (HVDC) transmission line.

The grounding method of a single polar DC system can lead to symmetric and asymmetric DC transmissions. Both kinds of grounding methods are widely used in practical projects. The symmetric DC transmission only employs one convertor to construct positive and negative poles and are more popular for voltage-source converters-high voltage direct current (VSC-HVDC) systems. A typical two-terminal VSC-HVDC system, as shown in Figure 1, is analyzed in this research and modeled on the platform of PSCAD/EMTDC. The midpoint of the DC supporting capacitor is grounded to form a symmetric DC transmission. Such a grounding method can reduce the insulation requirement of DC devices and avoid live currents in grounding loops under normal operation [27,28]. The bus voltage of VSC1 is controlled while the power of VSC2 is controlled.

Measuring units *M* are installed at the terminals of transmission lines to provide useful information for protection devices. The smoothing reactor *L* on both terminals of the transmission line can block high frequency components of the transient signal from convertors and thus reduce the influences of interferences beyond the protection zone of transmission lines. However, the transient interferences generated on transmission lines can still affect the protection judgement. To improve the reliability of DC line protections, the transients that can be detected by measuring units *M* should be discussed. Therefore, the most commonly encountered interference-lightning-is analyzed. The waveforms of faults and lightning interferences are compared and discussed.

### 2.2. Pole-To-Ground Fault

**Figure 2:**
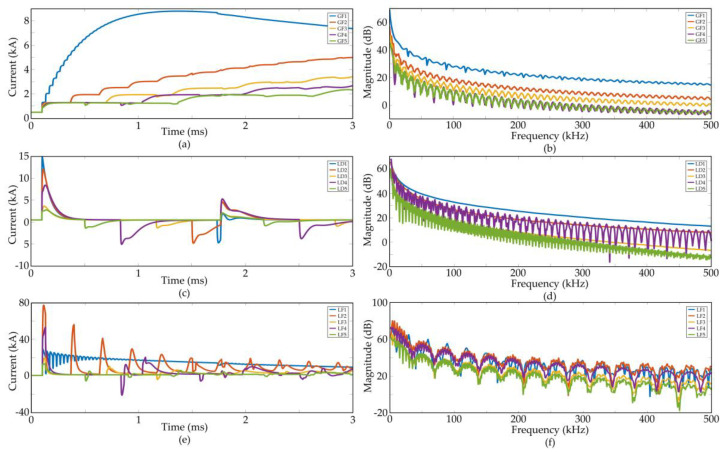
Comparison of different transient surges. (**a**) current waveforms of single-pole-to-ground faults (GFs); (**b**) frequency spectrums of GFs; (**c**) current waveforms of lightning disturbances (LDs); (**d**) frequency spectrums of LDs; (**c**) current waveforms of lightning faults (LFs); (**d**) frequency spectrums of LFs.

GF is actually a kind of short circuit fault. It is the most commonly encountered fault in practical HVDC transmission lines. For the symmetric grounding VSC-HVDC transmission system mentioned above, the fault transient procedure includes two stages: discharge of supporting capacitor and current feeding of alternating current (AC) sources [17]. In the first stage, the traveling wave produced by faults moves quickly along the transmission line. When it reaches the converter station, the supporting capacitor discharges quickly. Such discharge causes a large amplitude decrease of DC bus voltage and quick rising fault currents. As the bus voltage decreases to AC phase voltages, the AC sources begin to feed the fault circuit and the second stage starts. The diodes on each leg of convertor are commutated in an uncontrolled commutation mode, and the overall current tends to be steady. Figure 2a illustrates five typical waveforms of GF, and Figure 2b shows their frequency spectrums. The details of these five GFs are as follows: (1) *Z_g_* = 20 Ω, *L_f_* = 10 km; (2) *Z_g_* = 20 Ω, *L_f_* = 50 km; (3) *Z_g_* = 10 Ω, *L_f_* = 100 km; (4) *Z_g_* = 1 Ω, *L_f_* = 150 km; (5) *Z_g_* = 0.01 Ω, *L_f_* = 200 km. Here, *Z_g_* stands for the grounding impedance of fault, and *L_f_* denotes the distance of fault point. Although these waveforms look quite different in the time domain, they have similar frequency spectrums. All of their spectrums decrease smoothly from lower frequencies to higher ones. Small ripples that distribute equally along the frequency range can be found.

### 2.3. Lightning Transients

For long distance transmission, overhead lines are preferred in the aspect of economy and maintenance. But overhead lines are generally erected high away from the earth and located in open area, where lightning disturbance occurs. The lightning strokes can produce quick rising transients that are interferences in some cases or faults when the insulations are breakdown [29]. Therefore, recognition of the lightning transient surges has great significance to improve the reliability of traveling wave based protection.

The lightning stroke is actually a kind of discharge of electric charges between clouds and the earth. It can be modeled by a current source and injects current into the transmission systems. In the aspect of transmission line protection, lightning surges can be divided into two kinds: lightning disturbance (LD) and lightning fault (LF). The formation of these two transient surges are similar, but the results are different. Both LD and LF are caused by overvoltage that is produced by direct strokes or indirect strokes. For overhead transmission lines, the direct strokes that hit the bare conductor can generate large overvoltage and result in short-circuit of lightning surge arrestors. Although a large overvoltage is produced, the current is not so large due to the characteristic impedance of the transmission line (around several hundred ohms) and the operation of lightning protection devices at the line terminals. This kind of lightning stroke only generates electrical interferences. The lightning-caused traveling wave continuously refracts due to discontinuities at both ends of the line and eventually decays to zero [30]. The indirect strokes hit a point in the vicinity of the transmission lines-for example, the tower or shielding wire. If the lightning stroke current is high enough to cause tower-to-conductor flashover or shielding wire failure, LF occurs [31,32]. In this case, the induced currents in transmission line could be greater those due to LDs.

Generally, the lightning strike is modeled mathematically with a double exponential equation, as shown below [33,34].

(1) $$i\left(t\right)=AI_{L}\left(e^{-\alpha t}-e^{-\beta t}\right)$$

where *A* is the magnitude correction coefficient, *I_L_* is the amplitude of lightning strike, and *α* and *β* are the waveform coefficients and stand for the rising and falling time of the lightning impulse, respectively. Different lightning strikes are added to produce both LD and LF transients. The current waveforms of grounding fault and disturbances due to lightning stroke are simulated and displayed in Figure 2c,e, and their frequency spectrums are shown in Figure 2d,f.

Five LDs with different parameters are analyzed. The current lightning waveform 8/20 μs is used to simulate lightning strokes, and *α* = 7.714 × 10^4^ s, *β* = 2.49 × 10^5^ s, *A* = 2.331 [34]. The other parameters of the five scenarios are (1) *I_L_* = 15 kA, *L_f_* = 10 km; (2) *I_L_* = 15 kA, *L_f_* = 50 km; (3) *I_L_* = 5 kA, *L_f_* = 100 km; (4) *I_L_* = 15 kA, *L_f_* = 150 km; (5) *I_L_* = 5 kA, *L_f_* = 200 km. Different from the GF waveforms, which keep increasing, the LD waveforms are composed of successive impulses, and their magnitudes tend to be zero after a long duration. The frequency spectrum of LDs also attenuates gradually from lower frequency bands to higher ones. However, unlike those of GFs, the ripples of LD are greater and more irregular.

The parameters of LFs shown in Figure 2e are (1) *I_L_* = 30 kA, *L_f_* = 10 km, *Z_g_* = 10 Ω; (2) *I_L_* = 100 kA, *L_f_* = 50 km, *Z_g_* = 10 Ω; (3) *I_L_* = 30 kA, *L_f_* = 100 km, *Z_g_* = 20 Ω; (4) *I_L_* = 100 kA, *L_f_* = 150 km, *Z_g_* = 20 Ω; (5) *I_L_* = 30 kA, *L_f_* = 200 km, *Z_g_* = 10 Ω. The average amplitudes of LFs increase with time due to the grounding component. The spectrum energy of LFs also decrease with frequency, but the ripples are much greater than those of the other two kinds of transient surges.

Generally, the LF and GF currents increase gradually when the LD current oscillates around normal operating value. An easy method to distinguish fault and disturbance is to integrate the current waveforms, but this method usually needs tens of milliseconds, which is too long for a DC protection to make a judgement. Differences between the three kinds of transient surges can be revealed obviously in the frequency domain even though the time window of transient signal is only a few milliseconds. Appropriate selection of distribution features in frequency spectrum can help to discriminate the transients in extremely short duration.

## 3. FSE-Based Feature Extraction

### 3.1. Definantion of FSE

Entropy, a convenient tool for measuring the overall disorder of the system, has been effectively used in the field of signal processing [13,35]. If the frequency spectrum of any signal is considered as a system, its distribution can be characterized by entropy. In this paper, the frequency spectrum is generated by Fourier transform. The frequency spectrum is divided equally into *m* bands. The amplitude of the whole frequency spectrum is treated as a dataset, which is divided into *n* intervals to calculate the histogram of the frequency spectrum. The number of coefficients of *i*th (1 ≤ *I* ≤ *m*) band *x_i_* in *j*th (1 ≤ *j* ≤ *n*) interval is denoted as *x_ij_*, and the probability *p*(*x_ij_*) of *x_i_* is calculated according to Equation (2). The definition of FSE *H_i_* is shown Equation (3). Each frequency band can produce one entropy value. A FSE vector *H*_FSE_ with a size of *m* will be formed, as shown in Equation (4).

(2) $$p\left(x_{ij}\right)=\frac{x_{ij}}{\sum_{j=1}^{n}x_{ij}}$$

(3) $$H_{i}=-\sum^{}p\left(x_{ij}\right)\log_{b}\left(x_{ij}\right)\;\left(j=1,2,\ldots ,n\right)$$

(4) $$H_{FSE}=\left[H_{1}\;H_{2}\ldots H_{m}\right]$$

### 3.2. FSE Representation of Transient Surges

**Figure 3:**
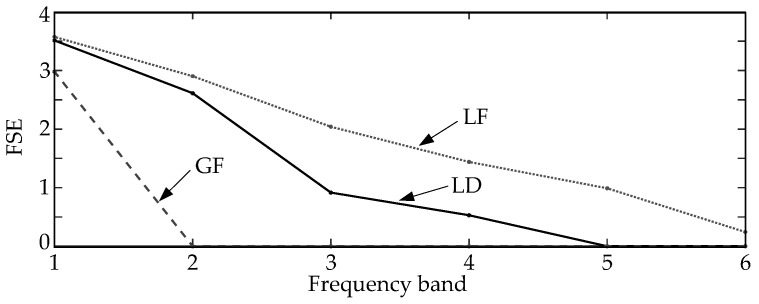
Frequency spectrum entropy (FSE) representation of different transient surges.

To illustrate the performance of FSE in representing different transient surges, the entropy vectors are analyzed under various scenarios and tested by the simulation model shown in Figure 1. The transient signal is sampled at a rate of 100 kHz, and the time window size is 3 milliseconds. Therefore, the data segment contains only 300 values (100 × 10^3^ sample per second × 3 × 10^−3^ millisecond). After Fourier transform, the frequency spectrum from the lowest frequency (except 0 Hz) to 50 kHz is divided into six frequency bands. Each frequency band has a range around 8.5 kHz. Figure 3 illustrates the FSEs of three different transient surges. It is clear that the entropy distribution of these FSE vectors are different from each other. The distribution of the frequency spectrum of GFs are more chaos in lower frequency bands, while the LDs and LFs have more even FSE distributions in whole frequency bands.

**Figure 4:**
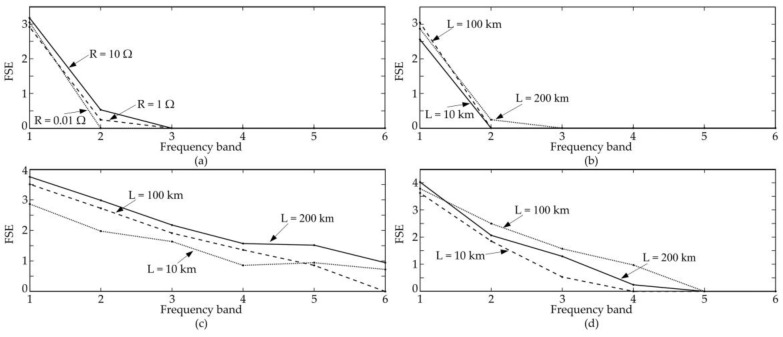
Effect of different factors on FSEs. (**a**) Effect of transition resistance of GFs; (**b**) effect of distance of GFs; (**c**) effect of distance of LFs; (**d**) effect of distance of LDs.

The reliability of FSE representation is also tested with different transition resistances and locations: transition resistance equals 0.01 Ω, 1 Ω and 10 Ω in grounding faults, and transients occur at 10 km, 100 km, and 200 km, respectively. As illustrated in Figure 4, the trends of FSE distribution vary slightly when parameters change.

## 4. SVM-Based Recognition Method

### 4.1. Foundamentals of SVM

SVM is a kind of machine learning algorithm based on statistical learning theory, Vapnik–Chervonenkis theory, and structural risk minimization. It has unique advantages in solving small sample, non-linear, and high-dimensional pattern recognition problems and has been widely used in the fields of pattern recognition and regression analysis [25,36].

SVM is a non-probabilistic binary linear classifier. Its main idea is to establish a classification hyperplane as the decision-making plane that maximizes its distance to the data [26]. For linearly separable data with *l* training samples, the design algorithm for an SVM is reduced to a convex optimization problem, as described in Equation (5), and its binary classification can be represented by Equation (6).

(5) $$\underset{w}{\min}\;\frac{1}{2}{\left\|w\right\|}^{2}\;\mathrm{subject}\;\mathrm{to}\;Y_{i}\left(w^{T}X_{i}+b\right)\geq 1\;\left(i=1,2,\ldots ,l\right)$$

(6) $$\underset{\alpha_{i}}{\max}\;-\frac{1}{2}\sum_{i=1}^{l}\sum_{j=1}^{l}Y_{i}Y_{j}\alpha_{i}\alpha_{j}\langle X_{i},X_{j}\rangle \;\mathrm{subject}\;\mathrm{to}\;\{\begin{array}{c} \sum_{i=1}^{l}Y_{i}\alpha_{i}=0\\ 0\leq \alpha_{i}\;\left(i=1,2,\ldots ,l\right) \end{array}$$

where *X_i_* ∈ *R^n^* is the *i*th feature, *Y_i_* ∈ {−1, 1} is the target label value (binary problem), *w* ∈ *R^m^* is the weight vector, $\alpha_{i}$ are the Lagrange coefficient, $\langle X_{i},X_{j}\rangle$ is the inner product of the input features vector $X_{i}$ and $X_{j}$, *b* is the bias term, and d(*w*, *b*) = *w*^T^*X_i_* + *b* = 0 defines the decision function (classification hyperplane). The weight vector *w* and the bias term *b* of decision function can be computed by Equations (7) and (8).

(7) $$w=\sum_{i=1}^{l}Y_{i}\alpha_{i}X_{i}$$

(8) $$b=\max w^{T}X_{i}+\min w^{T}X_{j}\;\mathrm{subject}\;\mathrm{to}\;\{\begin{array}{c} 1\leq i,j\leq l\\ Y_{i}=-1,Y_{j}=1 \end{array}$$

In practical applications, most kinds of data are not linearly separable in their original spaces. The original finite-dimensional space is then mapped to a much higher space to generate easier separation. The penalty parameter *C* and slack variables *ε_i_* are added to the decision function, as shown in Equation (9).

(9) $$\underset{w}{\min}\;\frac{1}{2}{\left\|w\right\|}^{2}+C\sum_{i=1}^{l}\varepsilon_{i}\;\mathrm{subject}\;\mathrm{to}\;\{\begin{array}{c} Y_{i}\left(w^{T}X_{i}+b\right)\geq 1-\varepsilon_{i}\\ 0\leq \varepsilon_{i}\;\left(i=1,2,\ldots ,l\right) \end{array}$$

Such optimization can be can be represented by a binary classification as shown in Equation (10).

(10) $$\underset{\alpha_{i}}{\min}\;\frac{1}{2}\sum_{i=1}^{l}\sum_{j=1}^{l}Y_{i}Y_{j}\alpha_{i}\alpha_{j}K\left(X_{i},X_{j}\right)-\sum_{j=1}^{l}\alpha_{j}\;\mathrm{subject}\;\mathrm{to}\;\{\begin{array}{c} \sum_{i=1}^{l}Y_{i}\alpha_{i}=0\\ 0\leq \alpha_{i}\leq C\;\left(i=1,2,\ldots ,l\right) \end{array}$$

To amplify the differences or the margins between data, every inner product $\langle X_{i},X_{j}\rangle$ that is related to the features vectors is replaced by a nonlinear kernel function, as shown in Equation (11).

(11) $$K\left(X_{i},X_{j}\right)≜\langle \varphi \left(X_{i}\right),\varphi \left(X_{j}\right)\rangle$$

Here, $K\left(X_{i},X_{j}\right)$ is the kernel function, φ is the nonlinear mapping. The use of kernel function allows the maximum-margin hyperplane to linearly separate data in transformed higher dimensional feature space. The kernel function is selected to suit the particular classification problem by testing the performance of kernel functions. The most commonly used kernel functions are listed in Table 1.

Though originated from the processing of binary classification, SVM can solve the problem of multi-classification by construction [37,38,39]. The construction of SVM can be divided into two categories: direct method and indirect method. The direct method is generally completed by modifying the objective function. Such method has a high computational complexity and is a bit of difficult in implementations. The indirect method is usually achieved by combining multiple binary classifiers. This solution is simple and easy to be used. “One-to-One” construction is one of the most commonly used indirect construction methods. It designs a SVM between any two types of samples, and determines the type of the unknown sample according to the category scores given by each SVM pair. This construction method greatly reduces the calculation complexity of each classification problem by increasing the number of binary classifiers, and the parallel computation of multiple classifiers improves the overall training speed and the classification accuracy. A “One-to-One” construction method is thus adopted to achieve multi-classification in this research.

### 4.2. Recognition Method

**Figure 5:**
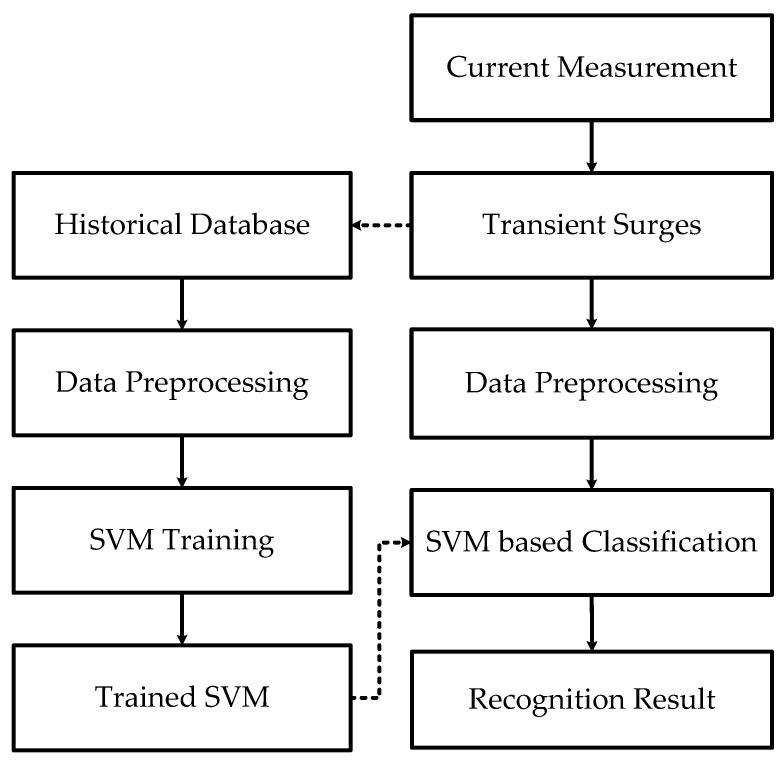
Flow chart of FSE- support vector machine (SVM)-based recognition method.

Combining the advantages of FSE and SVM, a transient surge recognition method is proposed. Its flowchart is shown in Figure 5. Four steps are included in this proposed method.

1. Signal acquisition

Since voltage is controlled in VSC-HVDC systems, the current measurements contain more transients than voltages ones and are thus employed in recognition. To avoid the influence from communications, only local measurements are used.

2. Data processing

A time window of 3 milliseconds is used to capture the starting part of the transient surges. Fourier transform is adopted to generate frequency spectrum. The FSE vector is calculated according to Equation (4).

3. SVM training

Training SVM is crucial for accurate discrimination of faults and disturbances. The structure of SVM is defined by using the “One-to-One” method.

4. Transient recognition

The trained SVM is tested with test samples and used for recognizing different kinds of transients.

## 5. Simulations

### 5.1. Simulation Model

A two-terminal point-to-point VSC-HVDC system, as shown in Figure 1, is built on the platform of EMTP/PSCAD. Double closed-loop PI control is designed to stabilize the bus voltage. The transmission capacity of this system is 200 MW and the DC bus voltage is ±200 kV. Coupled overhead transmission lines with frequency-dependent parameters are selected and the length of transmission line is 250 km. Transient current surges are simulated under different scenarios and with different parameters. The simulated transient sources are located equally along the transmission line with an interval of 10 km. The transition resistance of GFs and LFs varies randomly from 0.01 Ω to 20 Ω. The double exponential waveform, as demonstrated in Equation (1), is used to simulate lightning strokes. A typical current wave shape 8 μs/20 μs is adopted with ±10% variations in its parameters [34,40]. The amplitude of lighting strokes varies from 5 kA to 15 kA for LD, and from 30 kA to 100 kA for LF. The sampling rate is 100 kHz and the time window for surge capturing is 3 ms.

### 5.2. Data Processing

**Figure 6:**
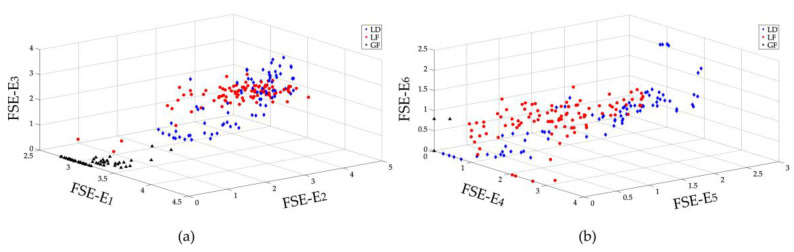
FSE feature map of different transient surges. (**a**) Space distribution of *E*_1_ vs. *E*_2_ vs. *E*_3_ (**b**) space distribution of *E*_4_ vs. *E*_5_ vs. *E*_6_.

The frequency spectrum of measured current surges are generated by Fourier transform. The whole frequency spectrum (frequency range (0 Hz, 50 kHz]) is divided into 6 frequency bands, and the total amplitude is divided into 30 intervals for FSE calculation. So, the FSE vectors have a size of 6, *E_FSE_* = [*E*_1_, *E*_2_, *E*_3_, *E*_4_, *E*_5_, *E*_6_]. For each kind of transients, 200 samples are collected, 100 samples of each kind are randomly selected to form the training sample set, and the rest 100 samples are used for testing. Since six-dimensional data cannot be demonstrated graphically, the *E_FSE_* vector is decomposed into two vectors: *E_FSE_*_1_ = [*E*_1_, *E*_2_, *E*_3_] that represents the lower frequency distributions and *E_FSE_*_2_ = [*E*_4_, *E*_5_, *E*_6_] that suggests the higher frequency distributions. Figure 6 shows all samples used for training: 100 samples for each kind of transient surges. The feature map or the space distributions of two decomposed vectors *E_FSE_*_1_ and *E_FSE_*_2_ are shown in Figure 6a,b, respectively.

As illustrated in Figure 6, the decomposed *E_FSE_*_1_ and *E_FSE_*_2_ vectors are nonlinearly separable in their three-dimensional spaces. In Figure 6a, the lower frequency features *E_FSE_*_1_ of three kinds of transient surges are mixed together, especially, the features of LD and LF. The FSE features *E_FSE_*_2_ of LD and LF in higher frequency range are close to each other. A few of the *E_FSE_*_2_ of GF mixed with those of LD. Therefore, the original feature vectors *E_FSE_* of the three kinds of samples are also linearly inseparable in six-dimensional spaces. A kernel function is thus needed to construct a decision surface in higher dimensional space.

### 5.3. SVM Training

As aforementioned, the “One-to-One” method is adopted in SVM training. Since there are 3 kinds of transient surges: LD, GF, and LF, three SVMs are employed to construct the following pairs: (i) 1st pair: LD-LF (SVM1), (ii) 2nd pair: LD-GF (SVM2), and (iii) 3rd pair: LF-GF (SVM3). The type of unknown transient can be determined by combining the results of each SVM. For example, an unknown transient surge can be determined to be LD only when both SVM1 and SVM2 produce LD classification results.

The selection of suitable kernel function is quite crucial for an excellent SVM classifier. The rate of correct recognition is used to evaluate the training performance. Four kinds of kernel functions-linear, polynomial, RBF, and sigmoid-are discussed. The K-fold Cross Validation (*K*-CV) is commonly used to choose parameter combinations to achieve highest classification accuracy, and avoid either over-learning or under-learning. The main idea of *K*-CV is to divide the original data into *K* groups, each of which includes both training and testing samples. The highest classification accuracy is taken as the objective function to determine the parameters of the SVM classifier. Here, *K* equals to 5 in this research. The mean recognition rates of five-fold cross validations are listed in Table 2.

As illustrated by Table 2, the kernel function RBF can produce higher recognition rates than others. The RBF kernel function is thus selected in FSE-SVM based recognition.

Other parameters, such as penalty parameter *C* and kernel function parameter γ, are tested by *K*-CV (*K* = 5). The common practice for parameter selection is to take the relevant parameters within a certain range. Both parameters are performed within a range from 0 to 1000. The values that give highest mean recognition rate are kept and used as the parameters of trained SVM. Table 3 lists all the selected parameters of each SVM and the overall mean recognition rates.

**Figure 7:**
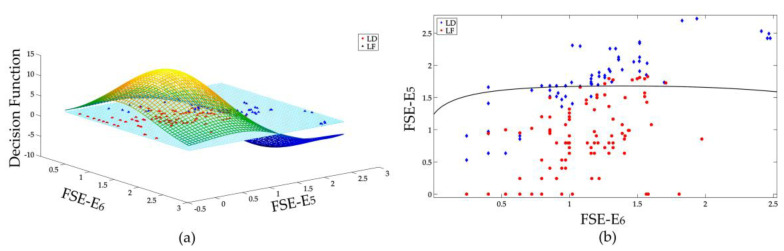
Binary classification of LD and LF, (**a**) decision surface in 3D space, (**b**) separation of LD and LF samples in 2D space.

**Figure 8:**
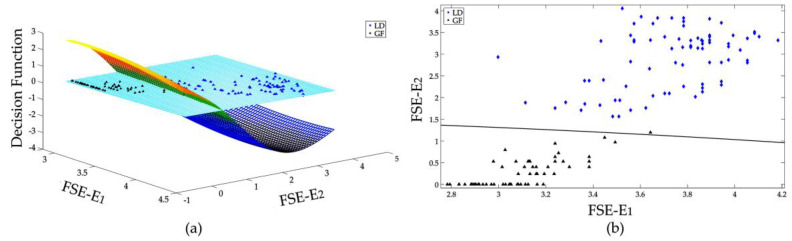
Binary classification of LD and GF, (**a**) decision surface in 3D space, (**b**) separation of LD and GF samples in 2D space.

**Figure 9:**
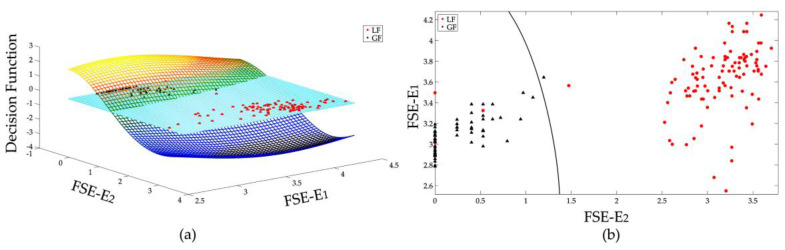
Binary classification of LF and GF, (**a**) decision surface in 3D space, (**b**) separation of LF and GF samples in 2D space.

To demonstrate the effectiveness of constructed SVMs, it is useful to illustrate the decision surface of each SVM. But for the proposed FSE vector which is 6 dimensional, only the decision surfaces of some selected features are displayed. The features that are more different from each other are selected for graphically illustrations. The decision surface of two distinguishing features of 3 SVMs that are used in this research are shown in Figure 7, Figure 8 and Figure 9, respectively.

Figure 7a displays the binary classification of LD and LF. As illustrated in Figure 6, the FSE features of LD and LF are quite similar. Two high frequency features: *E*_5_ and *E*_6_ are selected for illustration because their distributions are relatively far away from each other. The intersection of the decision function d(*w*, *b*) with the plane of features defines the optimal separation hyperplane, as shown in Figure 7b. By calculating the sign of decision surface d(*w*, *b*), the classification of LF and LD can be realized to some extent. As only two features are used for illustration, not all samples are effectively classified. With all 6 features, the classification results can be more effective.

Figure 8 and Figure 9 show the binary classifications of LD vs. GF and LF vs. GF, respectively, through only the feature *E*_1_ and *E*_2_. As shown in Figure 6, the large amplitude FSE features of GF gather more in the lower frequency range, which is different from those of transients caused by lightning strikes. As demonstrated by Figure 8b, the samples of LD and GF can be effectively classified with only *E*_1_ and *E*_2_. Also, most samples of LF and GF can be correctively distinguished with only two features *E*_1_ and *E*_2_. With the whole FSE vectors which includes six features, the GF can be effectively recognized from lightning-caused transients.

Hence, the SVM structure with appropriate kernel functions and parameters can effectively classify different kinds of transient surges.

### 5.4. Transient Recognition

The performance of the trained SVM is tested with test samples, and the recognition results are shown in Table 4. The GF can be discriminated with 100% recognition rates. Only four LF samples are classified to be LDs, and four LD samples are classified to be LFs. The overall recognition rate of proposed FSE-SVM based method is 97.33%, which shows great potential in protection application.

## 6. Comparisons

To demonstrate the effectiveness of proposed FSE-SVM-based method, the feature FSE and the classifier SVM are compared with existing popular methods-energy distribution and artificial neural network (ANN), respectively.

### 6.1. Comparison of Features

Energy distribution is one of the commonly used methods for frequency domain analysis of signals, which has the advantages of simple calculation and intuitive expression. However, the energy distribution heavily depends on the amplitude of transient surges in certain frequency band. The energy distribution defined by Equation (13) is used to characterize the frequency spectrum of transient surges [41]:

(12) $$E_{i}=\|c_{i}\|$$

(13) $$E=\left[E_{1}\;E_{2}\ldots E_{M}\right]$$

The frequency spectrum of transient surge is generated by Fourier transform. The whole frequency spectrum is divided into *M* bands. The energy *E_i_* of *i*th frequency band is the norm or square root of the sum of all Fourier coefficients *c_i_*. Here, *c_i_* stands for all of the coefficients in *i*th frequency band. The *M* energy value *E_i_* forms an energy distribution vector ‖E‖.

The energy distribution vector ‖E‖ is used as the feature, and the same SVM structure is adopted as classifier. The training procedure of SVM is as the same as the one discussed in Section 5. Table 5 shows the recognition results.

As demonstrated in Table 5, the recognition results of energy representations are lower than the proposed FSE based ones. The overall recognition rate is only 92.33%. The energy-based feature can effectively discriminate LF from other surges. However, it has difficulty in distinguishing faults and disturbances caused by lightning strokes. Only 90% of LDs can be correctly recognized. Among the misjudgments, four LDs are classified as GFs, and six LDs are regarded as LFs. Up to 13% of GF samples are classified as LFs. This might due to the energy attenuation and distortion of transient surges during propagation. The magnitude of the energy value varies a lot. However, the distribution, or disorder, of the frequency spectrum changes a little. When compared with energy based feature, the entropy based spectrum distribution is more effective in representing transient surges.

### 6.2. Comparison of Classifiers

Back-propagation (BP) ANN, which is a multi-feedforward network trained by error inverse propagation algorithm, is one of the widely used neural network models [42]. Different from a single SVM that can only distinguish two kinds of samples, a single BP ANN with proper design can realize recognition of multiple types. As the FSE dimension is six and the number of transient types is three, the number of neutrons of input and output layers of ANN are six and three, respectively. To achieve better performance, a lot of experiments are carried out to select the size of hidden layer, and six neutrons are finally chosen. Hyperbolic tangent function and linear function are selected to be the transfer function of hidden layer and output layer, respectively. The training function that based on gradient descent algorithm and dynamic adaptive learning rate is used.

The samples used in Section 5 are also characterized by FSE and recognized by ANN. Table 6 shows the recognition results of FSE-ANN based method.

As shown in Table 6, the overall recognition rate of FSE-ANN based method is a bit lower than that of FSE-SVM based one, which is 97.33%. As the same as SVM based recognition results, misjudgments occur for both LD and LF when ANN is adopted. But more samples are misjudged.

## 7. Conclusions

This paper proposed a FSE-SVM-based method to distinguish three kinds of most commonly encountered transient surges in HVDC transmission lines. The proposed method can generate effective recognition results and help improving the reliability of protections with relative lower sampling frequency (100 kHz) and extremely short data segment (3 ms). Simulations and comparisons between the energy-based feature and the ANN classifier demonstrate the FSE is stable in charactering the frequency spectrum of transient surges, and the “One-to-One” SVM structure is simple and effective for training and stable in performance. With training samples from precisely modeled simulation systems, the trained SVM can perform well and respond quickly in practical applications.

## Figures and Tables

**Table 1:** Typical kernel functions.

Type	Definition
linear	$K\left(X_{i},X_{j}\right)=X_{i}{}^{T}X_{j}$
polynomical	$K\left(X_{i},X_{i}\right)={\left(\gamma X_{i}{}^{T}X_{j}+r\right)}^{p},\gamma >0$
RBF	$K\left(X_{i},X_{j}\right)=\exp\left(-\gamma {\left\|X_{i}-X_{j}\right\|}^{2}\right),\gamma >0$
sigmoid	$K\left(X_{i},X_{j}\right)=\tanh\left(\gamma X_{i}{}^{T}X_{j}+r\right)$

Where γ,r and *p* are kernel parameters.

**Table 2:** Recognition rates of SVMs with different kernel functions.

SVM		Kernel Function			
		Liner	Polynomical	RBF	Sigmoid
Mean recognition rate	SVM1	84%	94%	95.5%	81%
	SVM2	100%	99.5%	100%	100%
	SVM3	100%	100%	100%	100%

**Table 3:** Optimal parameter selection results.

SVM	*C*	*γ*	Mean Recognition Rate
SVM1 (LD vs. LF)	5.66	1	95.5%
SVM2 (LD vs. GF)	0.004	0.25	100%
SVM3 (LF vs. GF)	0.004	0.5	100%

**Table 4:** Test results of FSE-SVM method.

Type	Recognition Rate	Misjudgment	Overall Recognition Rate
LD	96% (4/100)	4 (4 LF)	97.33% (292/300)
LF	96% (4/100)	4 (4 LD)	
GF	100% (100/100)	0	

**Table 5:** Test results of energy distribution and SVMs.

Type	Recognition Rate	Misjudgment	Overall Recognition Rate
LD	90% (90/100)	10 (4 GF, 6 LF)	92.33% (277/300)
LF	100% (100/100)	0	
GF	87% (87/1000)	13 (13 LF)	

**Table 6:** Test results of FSE and artificial neural network (ANN).

Type	Recognition Rate	Misjudgment	Overall Recognition Rate
LD	95% (95/100)	5 (5 GF)	91.67% (275/300)
LF	80% (80/100)	20 (20 LD)	
GF	100% (100/100)	0	
